# Safety and tolerability of cariprazine in the long-term treatment of schizophrenia: results from a 48-week, single-arm, open-label extension study

**DOI:** 10.1007/s00213-016-4450-3

**Published:** 2016-11-02

**Authors:** Suresh Durgam, William M. Greenberg, Dayong Li, Kaifeng Lu, Istvan Laszlovszky, Gyorgy Nemeth, Raffaele Migliore, Stephen Volk

**Affiliations:** 1 Allergan, Jersey City, NJ USA; 2 Gedeon Richter Plc, Budapest, Hungary; 3 Apostle Clinical Trials, Long Beach, CA USA

**Keywords:** Atypical antipsychotic, Dopamine antagonist, Cariprazine, Open-label, Safety, Schizophrenia

## Abstract

**Rationale:**

Cariprazine, a dopamine D_3_/D_2_ receptor partial agonist antipsychotic, demonstrated efficacy and tolerability in 6-week, randomized, placebo-controlled schizophrenia trials. Schizophrenia is a chronic disorder that requires continuous treatment; therefore, the long-term safety and tolerability profile of antipsychotic agents is an important factor in guiding clinician decisions.

**Objective:**

This single-arm, open-label extension study evaluated the long-term safety and tolerability of cariprazine in patients with schizophrenia.

**Methods:**

Patients enrolled in this study completed a 6-week, randomized, placebo- and active-controlled study and had responded (Clinical Global Impressions-Severity [CGI-S] ≤3; ≥20 % reduction in Positive and Negative Syndrome Scale [PANSS] total score) to treatment at the end of the lead-in study. Patients (*N* = 93) received flexibly dosed, open-label cariprazine (1.5–4.5 mg/day) for up to 48 weeks.

**Results:**

Approximately 50 % (46/93) of patients completed the 48 weeks of open-label treatment. The most common adverse events (AEs) were akathisia (14 %), insomnia (14 %), and weight increased (12 %). Serious AEs (SAEs) occurred in 13 % of patients; 11 % discontinued due to AEs. Mean changes in metabolic parameters were generally small and not clinically relevant. Mean body weight increased by 1.9 kg from the start of the lead-in study to the end of the extension study. There were no discontinuations associated with change in metabolic parameters or body weight. Long-term cariprazine treatment was not associated with prolactin elevation or clinically significant changes in cardiovascular parameters.

**Conclusions:**

In this 48-week, single-arm trial, open-label cariprazine (1.5–4.5 mg/day) treatment was generally safe and well tolerated with no new safety concerns associated with long-term treatment.

**Electronic supplementary material:**

The online version of this article (doi:10.1007/s00213-016-4450-3) contains supplementary material, which is available to authorized users.

## Introduction

Long-term treatment of schizophrenia remains a clinical challenge due to the high percentage of patients who experience recurring relapses over the course of illness (Robinson et al. 1999). A major factor for increased risk of relapse is lack of compliance or discontinuation of antipsychotic therapy (Robinson et al. 1999), which may be due to poor tolerability or loss of efficacy of the medication (Ascher-Svanum et al. 2010; Perkins 2002; Yamada et al. 2006).

While atypical antipsychotics generally result in fewer extrapyramidal symptoms (EPS) than first-generation agents, these compounds have the potential to induce adverse effects such as weight gain, metabolic changes, cardiovascular adverse events (AEs), hyperprolactinemia, and EPS (Leucht et al. 1999; Weiden 2007). The incidence of these adverse effects may differ significantly among currently available agents; therefore, understanding the tolerability profiles of currently available and new antipsychotics is an important component of effective clinical management of schizophrenia and should help guide treatment decisions. Although antipsychotic efficacy is thought to be principally mediated by D_2_ receptor occupancy (Nord and Farde 2011), currently available antipsychotics also have varying levels of affinity for additional neuroreceptors. These differences in receptor affinities may account for the variation among antipsychotics in efficacy, tolerability, and side effect profiles (Leucht et al. 2013). Antipsychotics with new receptor profiles that are generally well tolerated and show sustained efficacy across a broad range of symptoms are needed for improving long-term patient outcomes.

Cariprazine is an atypical antipsychotic that is approved in the USA for the treatment of schizophrenia and manic or mixed episodes associated with bipolar I disorder. It has a distinct pharmacological profile in that it shows potent dopamine D_3_ and D_2_ receptor partial agonism and preferential in vitro binding to D_3_ receptors (Kiss et al. 2010). In vivo, cariprazine demonstrates high occupancy of both D_3_ and D_2_ receptors at antipsychotic effective doses in rats (Gyertyán et al. 2011) and clinically active dose ranges in patients with schizophrenia (Girgis et al. 2016). This pharmacological profile differs from other atypical antipsychotics such as aripiprazole, clozapine, olanzapine, and risperidone, which have varying levels of in vitro affinity for D_3_ receptors but fail to show D_3_ receptor occupancy at clinically relevant doses (Caravaggio et al. 2014; Graff-Guerrero et al. 2009; Mizrahi et al. 2011). The D_3_ receptor is thought to play a role in mood and cognition (Gross and Drescher 2012), and cariprazine was developed based on the hypothesis that a compound that exhibits high in vivo binding affinity at both D_3_ and D_2_ receptors may confer benefits in treating the negative and cognitive symptoms associated with schizophrenia (Gyertyán et al. 2008; Joyce and Millan 2005; Kiss et al. 2008; Leriche et al. 2004; Zimnisky et al. 2013). Of note, cariprazine has demonstrated efficacy in clinical studies as monotherapy for bipolar I depression and as adjunctive therapy for major depressive disorder (Durgam et al. 2016a; Durgam et al. 2016c). Additionally, in a randomized, double-blind, active-controlled trial, cariprazine was significantly more effective than risperidone in treating predominant negative symptoms of schizophrenia and improving associated psychosocial impairment (Debelle et al. 2015).

Cariprazine also acts as an antagonist at serotonin 5-HT_2B_ receptors and as a partial agonist at 5-HT_1A_ receptors, with lower affinity for 5-HT_2A_, 5-HT_2C_, histamine H_1_, and adrenergic α_1_ receptors and negligible affinity at other receptors (Kiss et al. 2010). This distinct receptor binding profile may have beneficial implications for the cardiovascular (Leung et al. 2012), metabolic (Nasrallah 2008), sedative (Miller 2004), and hyperprolactinemia (Kapur and Seeman 2001) side effects that are associated with some antipsychotics.

The efficacy and safety of cariprazine (dose ranges 1.5–9 mg/day) were evaluated in short-term, randomized, placebo- and active-controlled phase IIb (Durgam et al. 2014) and phase III (Durgam et al. 2015; Kane et al. 2015) studies in patients with acute exacerbation of schizophrenia, as well as a double-blind, placebo-controlled relapse prevention study (Durgam et al. 2016b). This single-arm, open-label extension study was conducted to evaluate the long-term safety and tolerability of cariprazine 1.5 to 4.5 mg/day in patients that had completed 6 weeks of double-blind treatment during a phase IIb, placebo- and active-controlled lead-in study of cariprazine in patients with acute exacerbation of schizophrenia (Durgam et al. 2014).

## Methods

This was a multicenter, single-arm, open-label, flexible-dose, 53-week extension study (RGH-MD-17; NCT00839852) for outpatients who had completed 6 weeks of double-blind treatment with cariprazine (1.5, 3.0, or 4.5 mg/day), placebo, or risperidone 4.0 mg/day in a phase IIb lead-in study in patients with acute exacerbation of schizophrenia (RGH-MD-16; NCT00694707) (Durgam et al. 2014). This extension study was initiated 9 months after the initiation of the lead-in study; it was conducted between March 2009 and August 2010 at 40 study centers located in the USA (9), India (10), Malaysia (2), Russia (9), and Ukraine (10). It was designed in accordance with IHC and FDA GCP Guidelines; all participants provided written informed consent.

### Study design

The 53-week extension study comprised a no-drug screening period (3–7 days), 48 weeks of open-label treatment, and a 4-week safety follow-up period. Cariprazine was initiated at 1.5 mg/day, with 1.5-mg dose increases possible on days 2 and 3 depending on response and tolerability (based on investigator judgment) to a maximum dose of 4.5 mg/day. Decreases in dose by 1.5-mg/day decrements or a drug holiday of up to 3 days were allowed if there were tolerability issues. Patients may have been hospitalized during screening at the discretion of the investigator, and all patients were hospitalized during the first week of open-label treatment. After 1 week of open-label treatment, patients could be discharged and followed-up as outpatients or remain hospitalized for an additional week at the discretion of the investigator; patients could be rehospitalized at any time. Patients were evaluated weekly for the first 6 weeks of open-label treatment and biweekly for the duration of the study. Patients who completed 48 weeks of treatment or prematurely discontinued were evaluated for an additional 4 weeks during the safety follow-up period.

### Inclusion/exclusion criteria

Inclusion and exclusion criteria for the lead-in study have been previously described in detail (Durgam et al. 2014). Briefly, patients were 18 to 60 years of age and met the *Diagnostic and Statistical Manual of Mental Disorders*, Fourth Edition, Text Revision (DSM-IV-TR) criteria for schizophrenia with a current exacerbation of schizophrenia. Patients were required to have had the diagnosis for at least 1 year, with at least one previous psychotic episode, and have a Positive and Negative Symptoms Scale (PANSS) (Kay et al. 1987) total score of 80 to 120, inclusive (indicating moderate to severe symptoms), and a Clinical Global Impressions-Severity (CGI-S) (Guy 1976b) score ≥4 (moderately ill or worse).

Inclusion criteria for patients entering the extension study were the same as for the lead-in study, except that patients must have completed double-blind treatment in the lead-in study as outpatients, with CGI-S score ≤3 (mildly ill or better) and a ≥20 % reduction from lead-in study baseline in PANSS total score at the end of the lead-in study. Patients were also required to have normal physical examination, clinical laboratory, vital sign, and electrocardiogram (ECG) results or abnormal results that were not considered clinically significant. Designated caregivers were required to accompany outpatients at each visit or, in the case that they could not attend a visit, provide written documentation of patient’s study medication compliance.

Typical clinical trial exclusions were applied, including clinically significant, uncontrolled AEs or EPS during the lead-in study; pregnancy; significant risk for suicidal or violent behavior; various ophthalmology assessment criteria (e.g., history or current findings of ocular disease, history of intraocular surgery, laser treatment, or ocular trauma); and injection of a depot antipsychotic or electroconvulsive therapy since the lead-in study. Psychotropic medications were prohibited for the duration of the open-label study with the exception of SSRIs (citalopram, escitalopram, fluoxetine, fluvoxamine, or sertraline) and divalproex (with approval from the study physician). Zolpidem, zaleplon, eszopiclone, or chloral hydrate were permitted for insomnia; diphenhydramine, benztropine, or propranolol were permitted as EPS rescue medication; and lorazepam was permitted to control agitation, irritability, and hostility.

### Outcome assessments

Safety parameters included AEs, clinical laboratory parameters, vital signs, ECGs, and ophthalmologic examinations (e.g., Lens Opacities Classification System III). Suicidality was evaluated by the Suicidality Tracking Scale (STS) (Coric et al. 2009), an 8-item rating scale from 0 (“not at all”) to 4 (“extremely”) used to evaluate suicidality based on suicidal ideation and behavior. EPS was evaluated by the Barnes Akathisia Scale (BARS) (Barnes 1989), Abnormal Involuntary Movement Scale (AIMS) (Guy 1976a), and the Simpson-Angus Scale (SAS) (Simpson and Angus 1970). Efficacy was assessed by the PANSS and CGI-S scales; because the objective of the extension study was to evaluate the long-term safety and tolerability of cariprazine and there was no comparator group, efficacy assessments were not categorized as primary, secondary, or additional outcomes.

### Statistical analysis

All safety analyses were based on the safety population, which comprised all patients who took at least one dose of open-label cariprazine in this extension study; descriptive statistics were performed for safety parameters. The baseline of the lead-in study was used as the baseline for all safety parameters analyses (except for STS and ophthalmologic examination, which used the extension baseline since no lead-in baseline values were available). In this long-term safety study, use of the lead-in study baseline ensured that the reported safety outcomes were based on total cariprazine exposure. Mean changes in safety parameters were evaluated from baseline to end of study, with end of study values defined as the last available assessment during the open-label treatment period. AEs were considered treatment-emergent adverse events (TEAEs) if the AE started during open-label treatment and was not present before the first dose of double-blind treatment during the lead-in study or increased in intensity following the first dose of open-label treatment. AEs were analyzed separately for the safety follow-up period. Treatment-emergent parkinsonism was defined as an SAS score ≤3 at baseline and >3 postbaseline; treatment-emergent akathisia was defined as a BARS score ≤2 at baseline and >2 postbaseline.

All efficacy analyses were based on the intent-to-treat (ITT) population, which included patients from the safety population who had at least one efficacy assessment in this extension study. Efficacy analyses were performed using both the observed cases (OC) approach and the last observation carried forward (LOCF) approach; no inferential statistical analyses were performed for efficacy parameters.

## Results

### Patient disposition and demographics

**Fig. 1:**
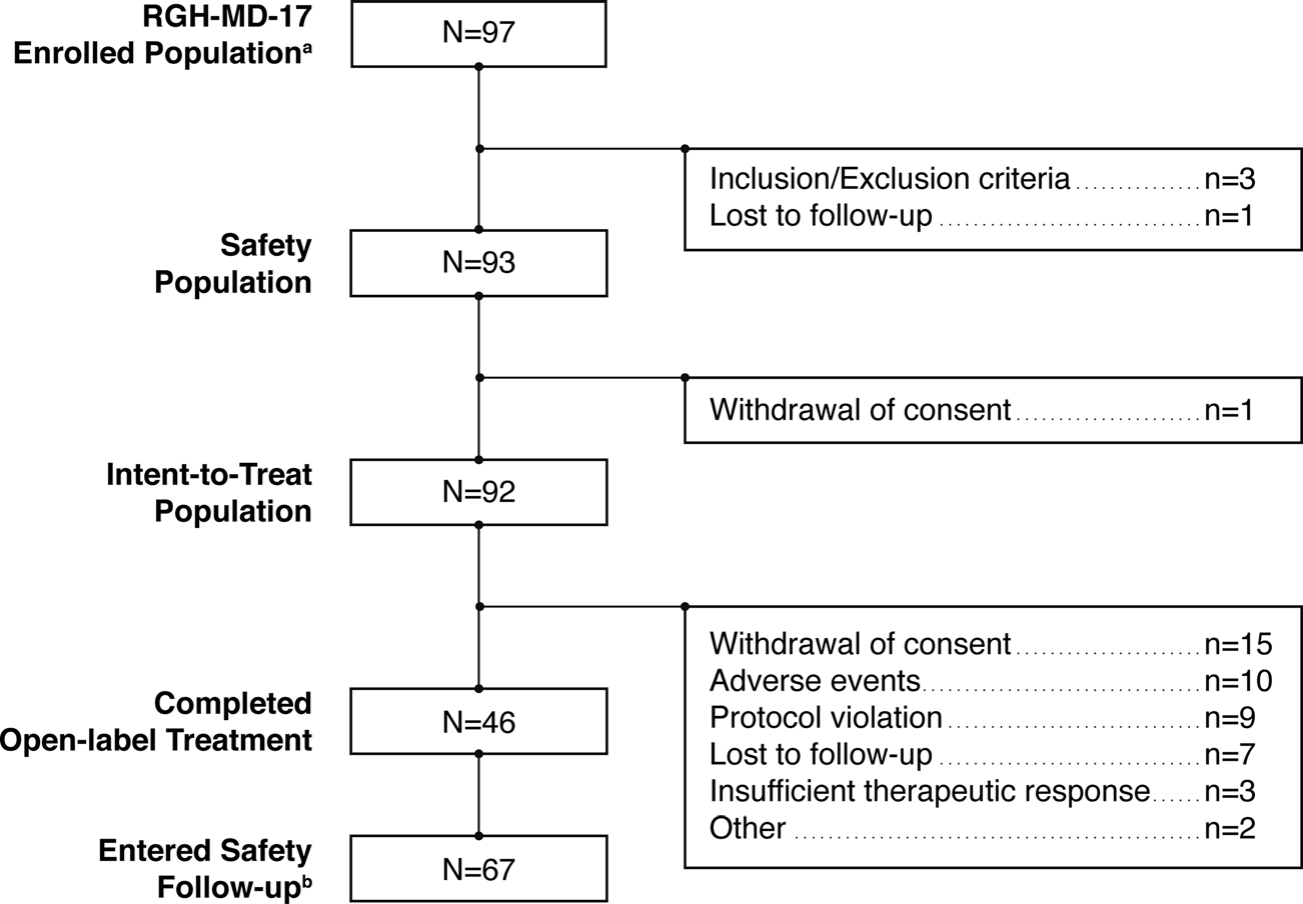
Patient populations and disposition. *Superscript a*: Since the extension study was initiated 9 months after initiation of the lead-in study, most completers from the lead-in study were not available for enrollment in the extension study. *Superscript b*: Includes patients who completed the study, as well as those who prematurely discontinued from the study but entered safety follow-up

Of the 464 patients who completed the lead-in study (RGH-MD-16), 97 patients enrolled in the extension study, and 93 received at least one dose of cariprazine (safety population, Fig. 1). Since the extension study was not initiated until 9 months after initiation of the lead-in study, a low number of completers were available to participate in the extension study. Of the 93 patients who received open-label cariprazine, over half (57.0 %) had received cariprazine in the lead-in study; 26.9 and 16.1 % had received risperidone and placebo, respectively. Per study protocol, patients were hospitalized for the first week of open-label treatment; 11 were rehospitalized due to clinical deterioration at a later point during the study. Demographics and characteristics of the safety population at baseline of the extension study are shown in Table 1. The mean duration of schizophrenia in the lead-in study was 11.6 years. A total of 92 patients in the safety population had at least 1 efficacy evaluation (ITT population), 46 patients completed the study, and 67 patients entered the safety follow-up period. Open-label discontinuations occurred in 47, 50, 54, 50, and 52 % of patients who had been randomized to placebo, cariprazine 1.5 mg/day, cariprazine 3.0 mg/day, cariprazine 4.5 mg/day, and risperidone 4.0 mg/day, respectively, in the lead-in study. The most frequent reasons for discontinuation during the open-label treatment period were withdrawal of consent (17.2 %) and AEs (10.8 %).

**Table 1:** Demographics and baseline characteristics (safety population)

	Cariprazine, *N* = 93
Lead-in study treatment group, *n* (%)	
Placebo	15 (16.1)
Cariprazine 1.5 mg/day	16 (17.2)
Cariprazine 3.0 mg/day	13 (14.0)
Cariprazine 4.5 mg/day	24 (25.8)
Risperidone 4.0 mg/day	25 (26.9)
Extension study demographics and baseline characteristics	
Age, mean (SD), years	34.4 (10.1)
Men, *n* (%)	63 (67.7)
Race, *n* (%)	
White	52 (55.9)
Black	11 (11.8)
Asian	30 (32.3)
Weight, mean (SD), kg	72.2 (17.7)
Height, mean (SD), cm	169.1 (10.7)
Waist circumference, mean (SD), cm	84.9 (11.2)
BMI, mean (SD), kg/m^2^	25.0 (4.5)

### Safety

#### Extent of exposure

The mean (SD) duration of cariprazine treatment was 221.7 (132.7) days; 46 patients (approximately 50 %) were exposed to cariprazine for at least 1 year. Patient-years of exposure (total treatment duration for all patients in days/365.25) was 56.4. Cariprazine 4.5 mg/day was the final dose for 70 % of the patients and was also the modal dose in 67.7 % of patients; 24.7 and 7.5 % of patients had modal daily doses of 3.0 and 1.5 mg/day, respectively.

#### Adverse events

Approximately 83 % of patients reported TEAEs, the most common of which (reported in ≥5 % of patients) are shown in Table 2. Approximately 80 % of patients reported an AE that was newly emergent (NEAEs, AEs that emerged or increased in severity after the first dose of open-label cariprazine). Most (72.9 %) were mild in intensity and 61 % were considered to be related or possibly related to cariprazine treatment. Most frequently reported TEAEs occurred early in treatment, and no unanticipated AEs emerged with continued long-term cariprazine therapy (Table 3). The only TEAEs that were considered treatment-related and occurred in ≥3 patients were joint stiffness, akathisia, and tremor.

**Table 2:** Summary of adverse events during open-label treatment (safety population)

	Cariprazine
	*N* = 93
	*n* (%)
Patients with any TEAE	77 (82.8)
Patients with NEAEs	74 (79.6)
Patients with SAEs	12 (12.9)
Deaths	1 (1.1)
Patients with AEs leading to premature discontinuation	10 (10.8)
Most frequent TEAEs (≥5 %)	
Akathisia	13 (14.0)
Insomnia	13 (14.0)
Weight increased	11 (11.8)
Headache	8 (8.6)
Nasopharyngitis	8 (8.6)
Agitation	7 (7.5)
Anxiety	7 (7.5)
Dizziness	7 (7.5)
Psychotic disorder	7 (7.5)
Schizophrenia	7 (7.5)
Tremor	7 (7.5)
Extrapyramidal disorder	6 (6.5)
Constipation	5 (5.4)
Diarrhea	5 (5.4)
Dyspepsia	5 (5.4)
Sedation	5 (5.4)
Somnolence	5 (5.4)

*AE* adverse event, *NEAE* newly emergent AE (emerged or increased in severity during extension study), *SAE* serious AE, *TEAE* treatment-emergent AE (emerged or increased in severity during extension study or lead-in study)

**Table 3:** Most frequent adverse events (≥5 %) by time to first occurrences (safety population)

	<6 weeks	6 to <12 weeks	12 to <24 weeks	24 to <36 weeks	36 to <48 weeks	≥48 weeks
Preferred term, *n* (%)	*n* = 93	*n* = 78	*n* = 66	*n* = 62	*n* = 50	*n* = 36
Akathisia	11 (11.8)	1 (1.3)	1 (1.5)	0	0	0
Insomnia	11 (11.8)	1 (1.3)	0	0	1 (2.0)	0
Weight increased	4 (4.3)	0	4 (6.1)	3 (4.8)	0	0
Headache	4 (4.3)	3 (3.8)	0	1 (1.6)	0	0
Nasopharyngitis	0	4 (5.1)	1 (1.5)	3 (4.8)	0	0
Agitation	7 (7.5)	0	0	0	0	0
Anxiety	5 (5.4)	1 (1.3)	0	1 (1.6)	0	0
Dizziness	4 (4.3)	2 (2.6)	1 (1.5)	0	0	0
Psychotic disorder	1 (1.1)	2 (2.6)	0	2 (3.2)	2 (4.0)	0
Schizophrenia	3 (3.2)	1 (1.3)	2 (3.0)	0	1 (2.0)	0
Tremor	5 (5.4)	1 (1.3)	1 (1.5)	0	0	0
Extrapyramidal disorder	5 (5.4)	0	1 (1.5)	0	0	0
Constipation	3 (3.2)	1 (1.3)	0	0	1 (2.0)	0
Diarrhea	4 (4.3)	0	0	1 (1.6)	0	0
Dyspepsia	4 (4.3)	0	0	1 (1.6)	0	0
Sedation	4 (4.3)	0	0	0	1 (2.0)	0
Somnolence	4 (4.3)	1 (1.3)	0	0	0	0

A total of 17 serious AEs (SAEs) were reported in 12 (12.9 %) patients during the open-label treatment period (Table 2), 5 of which were considered related or possibly related to treatment (exacerbation of schizophrenia [4 patients] and agitation/intentional overdose [1 patient]). The only SAEs that occurred in ≥2 patients were worsening of schizophrenia (4 patients) and worsening of psychotic disorder (2 patients). One death from suicide occurred after 327 days of treatment with cariprazine 4.5 mg/day. The event was not considered related to treatment; no trigger for the event was identified and the patient had no history of suicidal ideation or behavior. Ten patients (10.8 %) prematurely discontinued due to AEs (four AEs [headache, pneumonia, sedation, insomnia] and six SAEs [one completed suicide, one worsening of psychotic condition, and four worsening of schizophrenia]). Discontinuations due to AEs were distributed among patients who came into the study from the placebo (*n* = 2), cariprazine 3.0 mg/day (*n* = 2), cariprazine 4.5 mg/day (*n* = 4), and risperidone 4 mg/day (*n* = 2) treatment arms of the lead-in study. During the 4-week safety follow-up, three patients reported NEAEs, including two that were SAEs (worsening of schizophrenia and lower-limb fracture).

#### Clinical laboratory values and metabolic parameters

Mean changes from lead-in baseline in clinical laboratory values were generally small (Table 4). No clinically meaningful changes from baseline to the end of treatment were observed in liver function tests, and no patient met Hy’s Law criteria (alanine aminotransferase [ALT] or aspartate aminotransferase [AST] ≥3 × upper limit of normal [ULN] concurrent with total bilirubin ≥2 × ULN and alkaline phosphatase <2 × ULN). Mean prolactin and creatine kinase levels decreased from baseline to end-of-treatment period.

**Table 4:** Changes in clinical laboratory values (safety population)

	*N*	Cariprazine
Liver function, mean change (SD)^a^		
ALT, U/L	91	0.9 (17.0)
AST, U/L	91	−0.7 (15.0)
Total bilirubin, mg/dL	91	0.05 (0.28)
Alkaline phosphatase, U/L	90	−4.6 (33.5)
`Prolactin, mean change (SD)^a^		
Prolactin, ng/mL	80	−15.90 (26.73)
Creatine kinase, mean change (SD)^a^		
Creatine kinase, U/L	91	−56.44 (398.98)
Lipids and glucose, mean change (SD)^a^		
Total cholesterol, mg/dL	91	−5.02 (30.89)
Total LDL, mg/dL (calculated)	90	−4.63 (24.32)
Total HDL, mg/dL	91	1.93 (15.06)
Triglycerides, mg/dL	91	4.42 (113.27)
Fasting glucose, mg/dL	89	1.98 (24.14)
Clinically relevant shifts in lipids and glucose,^b^ *n* (%)		
Total cholesterol, normal/borderline (<240 mg/dL) to high (≥240 mg/dL)	82	0 (0.0)
Fasting LDL cholesterol, normal/ borderline (<160 mg/dL) to high (≥160 mg/dL)	81	0 (0.0)
HDL cholesterol, normal (≥40 mg/dL) to low (<40 mg/dL)	60	14 (23.3)
Fasting triglycerides, normal/borderline (<200 mg/dL) to high (≥200 mg/dL)	79	11 (13.9)
Fasting glucose, normal (<100 mg/dL) to high (≥126 mg/dL)	72	3 (4.2)
Fasting glucose, increase ≥10 mg/dL	89	26 (29.2)

*N* patients who had a lead-in baseline and ≥1 postbaseline measurement for the given parameter. *ALT* alanine aminotransferase, *AST* aspartate aminotransferase, *SD* standard deviation

^a^Mean changes are from lead-in baseline

^b^At end of open-label treatment

Mean total and LDL cholesterol levels were decreased from lead-in baseline to the end of the study, and small mean increases in HDL cholesterol were observed (Table 4). The incidence of clinically relevant lipid changes was low, with no patients shifting from normal/borderline levels of total (<240 mg/dL) or LDL (<160 mg/dL) cholesterol at baseline to high levels (total, ≥240 mg/d; LDL, ≥160 mg/dL) at the end of open-label treatment. Shifts from normal HDL cholesterol levels (≥40 mg/dL) at baseline to low levels (<40 mg/dL) at the end of open-label treatment occurred in 23.3 % of patients. Mean increases in triglycerides and fasting glucose were small and not clinically meaningful (Table 4). At the end of open-label treatment, 13.9 % of patients had shifted from normal/borderline to high levels of triglyceride (<200 to ≥200 mg/dL) and 4.2 % of patients with normal fasting glucose levels (<100 mg/dL) at baseline shifted to high levels (≥126 mg/dL). Less than one third (29.2 %) of patients had an increase in fasting glucose of ≥10 mg/dL. No metabolic change was reported as an SAE or led to study discontinuation.

#### Cardiovascular and physical findings

Mean changes in ECG parameters from lead-in baseline to endpoint were generally small and not clinically meaningful (Table 5). During the open-label treatment period, no patient had a QTcB or QTcF increase ≥60 ms or postbaseline value >500 ms. Changes in blood pressure and pulse rate were small (Table 5). Orthostatic hypotension (≥20 mmHg reduction in systolic blood pressure or ≥10 mmHg reduction in diastolic blood pressure while changing from the supine to standing position) was reported in approximately 25 % of patients during the open-label treatment period, which is similar to the rate reported by the placebo group in the lead-in study (23 %). Only two patients with orthostatic hypotension also reported TEAEs of dizziness; no other TEAEs suggestive of orthostatic hypotension were reported.

**Table 5:** Changes in vital signs, cardiovascular, and physical findings (safety population)

Assessment	*N* ^a^	Cariprazine
Blood pressure and pulse, mean change (SD)		
Systolic blood pressure, mmHg	93	0.6 (10.1)
Diastolic blood pressure, mmHg	93	0.2 (8.1)
Pulse, bpm	93	−1.3 (11.1)
Electrocardiogram, mean change (SD)		
Ventricular heart rate, bpm	91	−2.5 (15.6)
QRS interval, ms	91	1.2 (7.0)
PR interval, ms	91	0.8 (15.1)
QT interval, ms	91	3.2 (31.5)
QTcB, ms	91	−2.3 (21.2)
QTcF, ms	91	−0.2 (17.9)
Body weight, mean change (SD)		
Body weight, kg	93	1.87 (4.69)
Waist circumference, cm	79	3.09 (9.63)
PCS changes (≥7 %) in body weight, %		
≥7 % increase from baseline	93	33.3
≥7 % decrease from baseline	93	7.5

*QTcB* QT interval corrected for heart rate using the Bazett formula, *QTcF* QT interval corrected for heart rate using the Fridericia formula

^a^Patients in the safety population who had ≥1 postbaseline measurement for the given parameter

Mean change in body weight was +1.9 kg (1.5 kg in patients who received cariprazine in the lead-in study [*n* = 53] and 2.4 kg in both lead-in placebo patients [*n* = 15] and lead-in risperidone patients [*n* = 25]). Mean change from lead-in baseline to week 0 of the extension study (prior to open-label cariprazine treatment in this study) in the placebo, cariprazine, or risperidone lead-in groups was 0.9, 0.5, and 1.8 kg, respectively. Potentially clinically significant (PCS) weight gain (≥7 % increase from lead-in baseline) was experienced by 31 (33.3 %) patients, 5 of whom experienced weight increase of ≥15 %. Most (61 %) of the 31 patients who experienced ≥7 % weight increase were in the normal or underweight baseline BMI categories at baseline; 26 and 13 % were overweight and obese, respectively. PCS weight decrease (≤7 % decrease from lead-in baseline) was experienced by seven (7.5 %) patients.

#### Suicidality

Mean STS total score was unchanged during open-label treatment. As previously described, one male patient taking cariprazine 4.5 mg/day completed suicide. The patient had no history of suicidal ideation or behavior recorded on his STS scale, and the event was not considered to be treatment-related.

#### Extrapyramidal symptoms

Treatment-emergent parkinsonism (SAS total score ≤3 at baseline and >3 postbaseline) was reported in eight (8.6 %) patients, which is consistent with rates that were reported in the cariprazine and risperidone groups during the lead-in study (approximately 8–10 %). Treatment-emergent akathisia (BARS total score ≤2 at baseline and >2 postbaseline) was reported in 16 (17.2 %) patients, which is slightly higher than rates reported in the cariprazine and risperidone groups during the lead-in study (approximately 10–15 %). The most common EPS-related TEAEs during open-label treatment were akathisia (13 patients [14.0 %]), tremor (7 [7.5 %]), and extrapyramidal disorder (6 [6.5 %]); all were considered mild or moderate and no patient discontinued because of EPS-related TEAEs. Mean (SD) change from baseline to end of study in AIMS, BARS, and SAS scores was +0.2 (1.6), 0.0 (1.0), and −0.4 (1.7), respectively.

#### Ophthalmological examinations

There were no clinically significant changes in ophthalmologic parameters, including intraocular pressure, color discrimination, visual acuity, or lens opacity. Ocular TEAEs were reported in three (3.2 %) patients (blepharitis and corneal opacity [one patient], vision blurred [one patient], and cataract [one patient]). None of the ocular events were serious or led to premature discontinuation. The one reported adverse event of cataract involved a patient who completed 48 weeks of open-label treatment. The patient’s dose was adjusted early in the study and was eventually maintained on a dose of 3 mg/day for 285 days. No baseline ophthalmology assessments were conducted as this study was amended to include ophthalmology assessments after the patient had taken the first dose of study drug in both the lead-in and extension studies. There were no abnormal findings during the first ophthalmology examination on study day 211 or the examination on study day 227. On day 337 (the last visit of the open-label treatment period), an AE of cataract was reported when posterior subcapsular opacification increased from 0.1 to 2.7 units in the left eye. On day 374 and on day 395, left eye values for posterior subcapsular opacification decreased to 0.8 and 0.4 units, respectively. The cataract was considered resolved on day 374, 38 days after the patient’s last dose of open-label treatment. As the complete regression of a cataract is an unusual phenomenon, expert advice from an independent ophthalmologist was sought. The expert ophthalmologist determined that the reported AE was likely due to variability on the part of the examiner and did not represent an actual pathological event because (1) posterior subcapsular cataract of a magnitude to receive a 2.7 LOCS III grading rarely, if ever, occurs with any drug given for such a brief duration; (2) a 2.7 LOCS III grading level represents the observable expression of true tissue pathology and, as such, it is not evanescent and would not disappear over a period of weeks; and (3) drug-induced toxic cataract is a bilateral disorder.

### Efficacy

The primary objective of this study was to evaluate the long-term safety and tolerability of open-label cariprazine in adult patients with schizophrenia; therefore, no inferential statistical analyses were performed for the efficacy parameters. Decreases from the lead-in and extension baselines were observed in PANSS total score, PANSS Positive and Negative Subscale scores, and CGI-S scores at the end of open-label cariprazine treatment (Table 6 and Supplemental Table 1).

**Table 6:** Change in Efficacy Scores From Baseline to Week 48 (OC) (ITT Population)

	Cariprazine			
Efficacy measures	*N*	Mean (SEM) score	Mean (SEM) change from:	
			Lead-in baseline^a^	Extension baseline^b^
PANSS Total Score				
Lead-in baseline	92	97.3 (0.8)	—	—
Extension baseline	92	65.6 (1.4)	—	—
At Week 6	77	59.2 (1.3)	-37.9 (1.2)	-6.5 (1.0)
At Week 12	69	57.2 (1.4)	-39.9 (1.3)	-7.5 (1.2)
At Week 24	58	55.3 (1.4)	-42.1 (1.4)	-9.2 (1.0)
At Week 48	45	52.4 (2.0)	-44.8 (1.8)	-11.6 (1.4)
End of open-label treatment^c^	92	58.9 (1.6)	-38.5 (1.5)	-6.8 (1.3)
CGI-S Score				
Lead-in baseline	92	4.7 (0.1)	**—**	**—**
Extension baseline	92	3.0 (0.1)	**—**	**—**
At Week 6	77	2.7 (0.1)	-2.1 (0.1)	-0.3 (0.1)
At Week 12	69	2.7 (0.1)	-2.1 (0.1)	-0.3 (0.1)
At Week 24	58	2.6 (0.1)	-2.2 (0.1)	-0.4 (0.1)
At Week 48	45	2.4 (0.1)	-2.3 (0.1)	-0.6 (0.1)
End of open-label treatment^c^	92	2.7 (0.1)	-2.0 (0.1)	-0.3 (0.1)

^a^Lead-in baseline values were assessed 1 day prior to the first dose of double-blind treatment in the lead-in study.

^b^Extension baseline values were assessed at visit 2 (study week 0 following the screening period) of the open-label study.

^c^Last observation carried forward (LOCF) approach.

CGI-S indicates Clinical Global Impressions-Severity; PANSS, Positive and Negative Syndrome Scale; SEM, standard error of the mean.

## Discussion

This single-arm, open-label extension study supports the long-term safety and tolerability of cariprazine in patients with acute exacerbation of schizophrenia. Safety and tolerability outcomes from 48 weeks of open-label cariprazine treatment were consistent with those observed in the double-blind lead-in study (Durgam et al. 2014), with no new or unexpected findings. Although this study was designed to assess safety and tolerability, efficacy measures were collected, and it is noteworthy that there was no signal of worsening efficacy with continued cariprazine treatment for up to 1 year. These results are important given the chronic nature of schizophrenia that often necessitates continuous long-term treatment in order to manage symptoms and prevent relapse (Higashi et al. 2013).

The TEAE profile during long-term treatment was similar to that of the lead-in study. The most common TEAEs (≥10 % at any dose) during the 6-week lead-in study were insomnia, extrapyramidal disorder, akathisia, and constipation (Durgam et al. 2014). Similarly, the most common (≥10 %) TEAEs during the 48-week extension study were akathisia, insomnia, and weight increased. While SAEs occurred at a higher incidence in the extension study compared with the short-term lead-in study, the only SAEs that occurred in >1 patient during the extension study were related to the worsening of schizophrenia. Approximately 11 % of patients in the long-term study discontinued due to AEs, compared to 6–10 % of cariprazine patients, and 15 and 9 % of placebo and risperidone patients, respectively, during the lead-in study (Durgam et al. 2014).

Glucose dysregulation, lipid changes, and weight gain are commonly reported with atypical antipsychotic treatment and likely contribute to increased cardiovascular risk factors (Goff et al. 2005). At the end of the 6-week lead-in study, there were mean decreases in total cholesterol, LDL cholesterol, and triglyceride levels in the cariprazine group and mean increases in the risperidone group. At the end of the 48-week cariprazine treatment period, mean total and LDL cholesterol remained decreased from lead-in baseline and HDL levels were increased slightly from lead-in baseline. Mean triglyceride and glucose levels increased only slightly from lead-in baseline levels, indicating very little change over the course of the 1-year treatment period. Additionally, the percentage of patients that shifted from normal/borderline lipid or glucose values at baseline to high values at the end of open-label treatment was low. There were no clinically significant metabolic changes that were classified as an SAE or led to study discontinuation. These results suggest that long-term cariprazine treatment of up to 1-year duration was not associated with significant dyslipidemia or glucose dysregulation.

Increases in weight from lead-in baseline to end of extension study were greater in patients who received placebo (2.4 kg) or risperidone (2.4 kg) during the lead-in study compared with patients who received cariprazine (1.5 kg). However, mean changes from lead-in baseline to week 0 of the extension study were 0.5 kg in cariprazine lead-in patients and 0.9 and 1.8 kg in placebo and risperidone lead-in patients, respectively, suggesting that a substantial portion of the weight increase in the risperidone lead-in group may have occurred prior to initiation of open-label cariprazine treatment.

Increases of at least 7 % of baseline body weight occurred in approximately one third of cariprazine-treated patients during the open-label treatment period; conversely, approximately 8 % of patients experienced clinically significant decreases in body weight. There were no SAEs or discontinuations associated with body weight changes. Clinically significant increases in weight were most frequent in patients who were classified as underweight or normal BMI at baseline (19 of the 31 incidences) and least frequent in patients classified as obese at baseline (4 incidences).

The incidence of akathisia as a TEAE in this study was similar to that observed in previous short-term studies of cariprazine (Durgam et al. 2015; Kane et al. 2015) and comparable with rates associated with other second-generation agents (Kane et al. 2009). All incidences of akathisia in the study were mild or moderate in intensity, and mean BARS scores were unchanged at the end of treatment. No EPS-related TEAE resulted in premature discontinuation from the study.

Long-term cariprazine treatment was not associated with prolactin elevation, high levels of sedation or somnolence, or clinically significant changes in cardiovascular parameters. The distinct receptor binding profile of cariprazine may contribute to the differential safety and tolerability profile relative to other antipsychotics. Cariprazine has low affinity for adrenergic and cholinergic receptors and has low potential for inhibiting hERG channel activity, features which may reduce the risk of cardiovascular adverse effects, including orthostatic hypotension and QT prolongation (Leung et al. 2012). Similarly, the low affinity of cariprazine for histaminergic receptors may explain the lack of sedative effects (Miller 2004) at therapeutic doses. Unlike some antipsychotics with high affinity for dopamine D_2_ receptors, cariprazine did not result in increased prolactin elevation; this finding suggests that partial agonist activity of cariprazine at dopamine D_2_ receptors does not impair tuberoinfundibular pathway function (Kapur and Seeman 2001).

Ophthalmology testing was initiated in the cariprazine clinical development program in response to ocular findings observed in the nonclinical program (i.e., cataract formation in dogs and melanin binding in the mass balance study in pigmented rats). No evidence of retinal toxicity or lenticular changes of clinical significance has been found in short- or long-term cariprazine clinical studies and, consistent with these findings, no clinically significant ophthalmologic changes were observed in this long-term cariprazine study.

This study was limited by its open-label design and lack of placebo or active comparator arm. In addition, as participants in this study were required to complete a previous 6-week placebo- and active-controlled study, the population in this study may reflect patients who responded to and tolerated initial treatment and may not be generalizable to all patients. Further, the extension study was initiated 9 months after the initiation of the lead-in study; therefore, patients who were enrolled early into the lead-in study may not have been available to enroll in the extension phase. While all patients underwent a 1-week washout period, differences in lead-in study treatments (placebo, cariprazine, or risperidone) may confound interpretation of some results as changes in safety parameters were assessed relative to the baseline of the lead-in study. Although efficacy measures were collected, it is difficult to interpret efficacy in a long-term, single-arm, open-label trial due to the lack of a control group and the influence of study dropouts on mean changes in rating scale values. Finally, this study assessed the long-term safety and tolerability of cariprazine at doses of 1.5 to 4.5 mg/day; investigation of the long-term safety of the highest recommended dose of cariprazine (6 mg/day) is needed.

In conclusion, this study shows that open-label treatment with cariprazine at flexible doses ranging from 1.5 to 4.5 mg/day was generally safe and well tolerated for up to 1 year without any apparent loss of efficacy. The results of this long-term extension study are similar to those observed in the short-term double-blind lead-in study and indicate that there are no new safety concerns associated with open-label long-term treatment. These findings support the long-term use of cariprazine in patients with schizophrenia.

## Electronic supplementary material

Table S1 (DOCX 527 kb)
